# Early Root Herbivory Impairs Arbuscular Mycorrhizal Fungal Colonization and Shifts Defence Allocation in Establishing *Plantago lanceolata*

**DOI:** 10.1371/journal.pone.0066053

**Published:** 2013-06-19

**Authors:** Alison E. Bennett, Anna M. Macrae, Ben D. Moore, Sandra Caul, Scott N. Johnson

**Affiliations:** 1 The James Hutton Institute, Invergowrie, Dundee, United Kingdom; 2 College of Life Sciences, University of Dundee, Dundee, United Kingdom; 3 The James Hutton Institute, Craigiebuckler, Aberdeen, United Kingdom; Nanjing Agricultural University, China

## Abstract

Research into plant-mediated indirect interactions between arbuscular mycorrhizal (AM) fungi and insect herbivores has focussed on those between plant shoots and above-ground herbivores, despite the fact that only below-ground herbivores share the same part of the host plant as AM fungi. Using *Plantago lanceolata* L., we aimed to characterise how early root herbivory by the vine weevil (*Otiorhynchus sulcatus* F.) affected subsequent colonization by AM fungi (*Glomus* spp.) and determine how the two affected plant growth and defensive chemistry. We exposed four week old *P. lanceolata* to root herbivory and AM fungi using a 2×2 factorial design (and quantified subsequent effects on plant biomass and iridoid glycosides (IGs) concentrations. *Otiorhynchus sulcatus* reduced root growth by c. 64%, whereas plant growth was unaffected by AM fungi. Root herbivory reduced extent of AM fungal colonization (by c. 61%). *O. sulcatus* did not influence overall IG concentrations, but caused qualitative shifts in root and shoot IGs, specifically increasing the proportion of the more toxic catalpol. These changes may reflect defensive allocation in the plant against further attack. This study demonstrates that very early root herbivory during plant development can shape future patterns of AM fungal colonization and influence defensive allocation in the plant.

## Introduction

It is now accepted that terrestrial plants mediate interactions between organisms associated with them, often shaping ecosystem processes and community structure [1], [2]. In particular, interactions between herbivorous insects and plant-associated fungi have become especially well studied, with numerous examples involving plant pathogens [3], [4], endophytes [5], [6] and symbionts (e.g. [7], [8]). Within the last group, plant-mediated effects of arbsucular mycorrhizal (AM) fungi have been shown to be beneficial, detrimental or neutral for herbivore fitness (reviewed in [9], [10]), and the evolutionary outcomes have been explored [7]. In particular, it has been suggested that AM fungal-induced increases in defensive chemical concentrations lead to an increased selective advantage for plants in plant-herbivore interactions [7]. Surprisingly, current understanding of the reverse interaction (*i.e.* the effect of insect herbivores on AM fungi) is largely derived from studies that consider the indirect effects of above-ground insect herbivores on AM fungi, despite the two being spatially separated [9], [11], [12]. Given that root herbivores and AM fungi exploit the same part of the plant, either simultaneously or at different times, it seems likely that root herbivory influences AM fungal colonization. Despite this, very few studies have addressed how root herbivores and AM fungi interact, and the outcomes of these studies are mixed. For instance, AM fungi can negatively (e.g. [13], [14]) or positively (e.g. [15]) affect root herbivores, whereas chewing root herbivores may stimulate [15], [16], or have no effect on (e.g. [13], [14]) AM fungal colonization.

In many ecosystems, root herbivores have been shown to influence the community composition of plants, above-ground herbivores and higher trophic groups [17], [18]. These effects are likely related to the propensity of root herbivores to induce systemic changes in secondary metabolites in plants more frequently than shoot-feeding insects [19], [20]. Indeed, low levels of root herbivory can lead to defensive ‘priming’ in the plant, resulting in reallocation of resources to defend against future herbivory [19], [21], [22]. AM fungi can cause similar changes in secondary metabolites throughout the plant [11], [23], but no studies have yet considered the combined effect of root herbivory and AM fungi on plant secondary metabolites or whether their effects are additive, synergistic or antagonistic.

This study set out to test how root herbivory by the vine weevil (*Otiorhynchus sulcatus* F.) feeding on *Plantago lanceolata* L. affected AM fungal (*Glomus* spp.) colonization and how this ultimately influenced plant growth and subsequent induction of secondary metabolites. *Plantago lanceolata* is a frequently used model system for investigating the effects of AM fungal colonisation (e.g. [24], [25], [26]) and herbivory (e.g. [27], [28]–[31]), and their interaction (e.g. [23], [32], [33]). *Otiorhynchus sulcatus* is a generalist root herbivore, known to attack a broad range of plant species, including *P. lanceolota* [34]. The main group of secondary metabolites in *P. lanceolota* are the iridoid glycosides [35], [36], which are dominated by two compounds in particular: aucubin and catalpol [37]. Catalpol is a derivative of aucubin and more metabolically costly [35], [36]. These compounds can have defensive roles both against herbivores [31], [38] and microbes [39]. Iridoid glycoside concentrations have been shown to vary with AM fungal species in shoots of *P. lanceolata* [23], [40], [41] as well as with nematode root herbivory [42]. The combined effects of root herbivory and AM fungi on iridoid glycosides are unknown.

Using a factorial experiment, *P. lanceolata* plants were treated with either AM fungal inocula or sterilized AM fungal inocula, and then either exposed to a brief period of root herbivory by *O. sulcatus* or not exposed. The effects of *O. sulcatus* herbivory and AM fungi (acting alone and in combination) on plant growth and iridoid glycoside (aucubin and catapol) concentrations in the roots and shoots were then determined, as was the effect of initial *O. sulcatus* herbivory on AM fungal colonization. Based on previous research in other systems we hypothesized that early root herbivory by *O. sulcatus* would reduce plant growth, increase AM fungal colonization [15], [16], and systemically increase total concentrations of iridoid glycosides in mature *P. lanceolota* [19], [20]. Root herbivory has been hypothesized to increase root exudates to which AM fungi respond resulting in an increase in AM fungal abundance in roots [15], [16]. In addition, we hypothesized that there would be a negative correlation between iridoid glycoside concentrations in the root, and AM fungal root colonization [40].

## Materials and Methods

### Study system

*Plantago lanceolata* seeds were collected from a well-established population on the grounds of the James Hutton Institute, Invergowrie, Scotland. *Otiorhynchus sulcatus* larvae used in this experiment were offspring of adults collected at night from plants and bushes surrounding the aforementioned *P. lanceolata* population. Adult vine weevils were stored in a controlled environment room at 19°C with 16∶8 hours light∶dark in Petri dishes. Each petri dish contained one large strawberry leaf (cut at the base of the leaf from a glasshouse stock of strawberry plants) as well as two discs of standard laboratory paper towels (equal to the diameter of the petri dish) moistened with tap water [43]. AM fungal inoculum was created by combining equal volumes of inocula (each containing at least 10 spores per gram in addition to hyphae and colonized roots) of two *Glomus* species cultured separately on *P. lanceoalata*: *Glomus* sp. and *Glomus intraradices* (INVAM #FR121) (sold commercially by Agrauxine, Saint Evarzec, France).

### Experimental Protocol

The experiment followed a 2×2 factorial design consisting of two AM fungal treatments (a mixture of *Glomus* sp. and *G. intraradices* or a sterilized mixture of both species) and two vine weevil treatments (presence or absence). Each treatment was replicated ten times giving a total of 40 individually potted plants.

*Plantago lanceolata* seeds were germinated on a mist bench in steam sterilized coir (Roffey Limited, Dorset, UK) in a glasshouse for four weeks. Soil (heat-sterilized soil (loam); B&Q, UK) and sand were steam-sterilized twice, homogenized, and mixed 1∶1 to improve drainage and reduce the nutrient content of the soil (thereby ensuring an environment in which AM fungi were unlikely to be parasitic). Deepots (Cat #D40H, Stuewe & Sons, Grants Pass, OR, USA) were filled by adding 100 ml of the sterilized mixture of sand and soil to the pot, followed by a mixture of 300 ml of sterile sand: soil and 100 ml of inoculum (steam-sterilized or unsterilized *Glomus* sp. and *Glomus intraradices*), and topped with 100 ml of sterile sand: soil for a total of 600 ml. Inclusion of sterilized AM fungal inoculum controlled for any change in the structure or nutrient content of the soil resulting from the addition of the inoculum itself. We used this design because we wanted to explore only the role of AM fungi on plant responses to root herbivores, and we plan to incorporate more complex soil communities in future experiments. A single, randomly-chosen seedling was transplanted into each pot. Plants from each treatment combination were placed into two spatial experimental blocks located at different ends of a bench in the same greenhouse room.

Three days after the seedlings had been transplanted, 20 one-day-old *O. sulcatus* eggs were placed around each plant (c. 0.5 cm below the soil surface) in the twenty pots assigned for weevil addition (10 with sterilized and 10 with unsterilized AM fungal inocula). This approximates the typical egg density that may be laid by a colonising adult *O. sulcatus* under the prevailing environmental conditions at the site, as previously reported [44]. In a separate experiment we added vine weevil eggs to plants of the same age grown in the same soil and pots and assessed vine weevil numbers after one week by emptying pots and examining their contents under a dissecting microscope. This “extraction method” allowed us to assess whether eggs had hatched and larvae (generally a few mm long) were alive. No white vine weevil larvae were present eight days after eggs were added, but hatched eggs were observed in the soil. This indicates that likely vine weevil eggs hatched, fed, and larvae expired within eight days of addition to our experimental pots. As a result we are confident that the herbivory treatment was short in duration (especially given that in the field vine weevils feed on roots for seven months), but the results also clearly demonstrate that the effect of this herbivory on the plants was significant. The duration of herbivory was not deliberately manipulated, but rather was determined by the food resource available and the response of the herbivores. Two weeks following the addition of vine weevil eggs (17 days after transplantation) the length of each plant leaf was measured and all lengths added together to create the variable “total leaf length” every two weeks for 10 weeks. Two weeks after the addition of weevil *O. sulcatus* eggs, plants were fertilized with 200 ml of a simple 20-0-20 NPK solution (1 mM NH_4_NO_3_ and 5 mM KNO_3_), and this was then repeated every two weeks for the duration of the experiment.

While we demonstrated that *O. sulcatus* larvae could not survive longer than one week on *P. lanceolota* plants of this size, we also confirmed that no weevils had completed the larval stage (and emerged above-ground as adults) by isolating deepots in water-filled moats. Larvae do not readily come to the soil surface or move between pots, even when under duress (e.g. [45]), but any emerging adults would have fallen into the moats and been easily identified. The moats also prevented any incidental *O. sulcatus* climbing onto or between pots and foliage showed no indication of weevil herbivory.

Plants were harvested 11 weeks after transplantation. Total summed leaf lengths were recorded on the day of harvest prior to the removal of plant tissue. Above-ground tissues were removed by cutting at the base of the rosette and were flash-frozen in liquid nitrogen. Root systems were washed and care was taken to search for any vine weevil larvae present within pots before flash freezing roots. All samples were then freeze-dried, their dry weights recorded, and stored at −80°C.

AM fungal colonization was assessed by removing a random sample (<0.1 g dry weight) from each root system after freeze-drying, placing these samples into tissue cassettes, clearing with KOH, and staining with trypan blue. Slides of stained roots were analyzed using the gridline intersect method [46] at ×40 magnification to score AM fungal structures, including hyphae, arbuscules, vesicles and spores in at least 100 fields of view per root system.

### Iridoid glycoside analysis

Shoot and root material were analyzed for the iridoid glycosides aucubin and catalpol following a variation of the protocol of Bowers and Stamp [37] and Gardner and Stermitz [47]. Shoot and root tissues were ground using a ball mill (Mini-BeadBeater 8, BioSpec, Bartlesville, OK, USA). Sub-samples of ground shoot (0.025 g) and ground root (0.035 g) samples from plants with sufficient available biomass were extracted in methanol (HPLC grade, Rathburn Chemicals, Walkerburn, Scotland), solid particles were removed by filtration, and the extracts were evaporated at 60°C under a stream of nitrogen. One ml of an internal standard (phenyl-β-D-glucopyranoside) (0.5 mg/ml) was added to each dry sample followed by 3.0 ml dH_2_O, and these solutions were then subjected to three washes with pure diethyl ether to remove chlorophyll and other compounds before drying as previously. Dried samples were redissolved in 1.0 ml methanol and two replicate 100 µl aliquots were dried again before being derivatised using a 1∶4 mixture of N,O-bis(trimethylsilyl)trifluoroacetamide (BSTFA, Sigma Aldrich, UK) and pyridine before 1 µl was injected into an Agilent 7890A gas chromatograph with multimode injector operating in split mode at 275°C with a split ratio of 30∶1. A Restek (Thames Restek, Buckinghamshire, UK) Rxi-1ms column (length 30 mm, internal diameter 0.25 mm, coat thickness 0.25 µm) was used with helium carrier gas delivered at 1.5 ml min^−1^. The oven conditions were 200°C at injection, held for 1 min, before increasing at 20°C min^−1^ to 260°C, held at this temperature for 6 min, then increased at 20°C min^−1^ to 320°C, was and held for 10 min, giving a total run time of 23 min. The detector used was a flame ionisation detector which was operated at 320°C using nitrogen as a make-up gas. Peaks were identified by reference to the retention time of derivatized authentic aucubin and catalpol standards (Sigma Aldrich, UK).

### Statistical Analyses

Data were analyzed using four statistical tests. First, to test for the effects of block, experimental treatments (AM fungal inocula and vine weevil herbivory) and their interaction on total plant biomass, root biomass, root and shoot total iridoid glycoside concentrations, and the ratio of catalpol to aucubin in roots and shoots, we conducted an analysis of variance (ANOVA) in the general linear models procedure of SAS 9.2 (SAS Insitute, Cary, NC). Plant biomass, root biomass, root and shoot total iridoid glycosides, and catalpol∶aucubin in roots and shoots were log-transformed. Proportion of root length colonized by AM fungi was analyzed using ANCOVA in the general linear models procedure of SAS 9.2 (SAS Insitute, Cary, NC). Proportion of root length colonized by AM fungi was arcsin square root-transformed to meet the assumptions of normality. Root biomass was included as a covariate in the analysis of AM fungal colonization to control for variation in root length or biomass that could influence measures of proportional colonization. Second, we determined whether total leaf length was an acceptable proxy for plant biomass by testing for a correlation between the log-transformed variables of final total leaf length and total plant biomass. Third, we examined which factors had the greatest influence on plant biomass throughout the experiment. To test this we conducted a repeated measures ANOVA in the general linear models procedure of SAS 9.2 (SAS Insitute, Cary, NC) on the five measures of total leaf length (used as proxies of plant biomass at five different time points) spanning the length of experiment. This test confirmed that changes in plant biomass were correlated with *O. sulcatus* presence. Finally, to test whether AM fungal colonization was negatively correlated with root iridoid glycoside concentration or composition as previously observed by De Deyn et al [40], we conducted separate correlation analyses for plants in the two vine weevil treatments that were also in the AM fungal treatment in the correlation procedure of SAS 9.2 (SAS Institute, Cary, NC) between the log of the concentration of total iridoid glycosides or the log of the ratio of catalpol∶aucubin in roots and the arcsin square root-transformed proportion of root length colonized by AM fungi. SAS code for all the analyses is included in File S1.

## Results

### Plant Biomass

**Figure 1:**
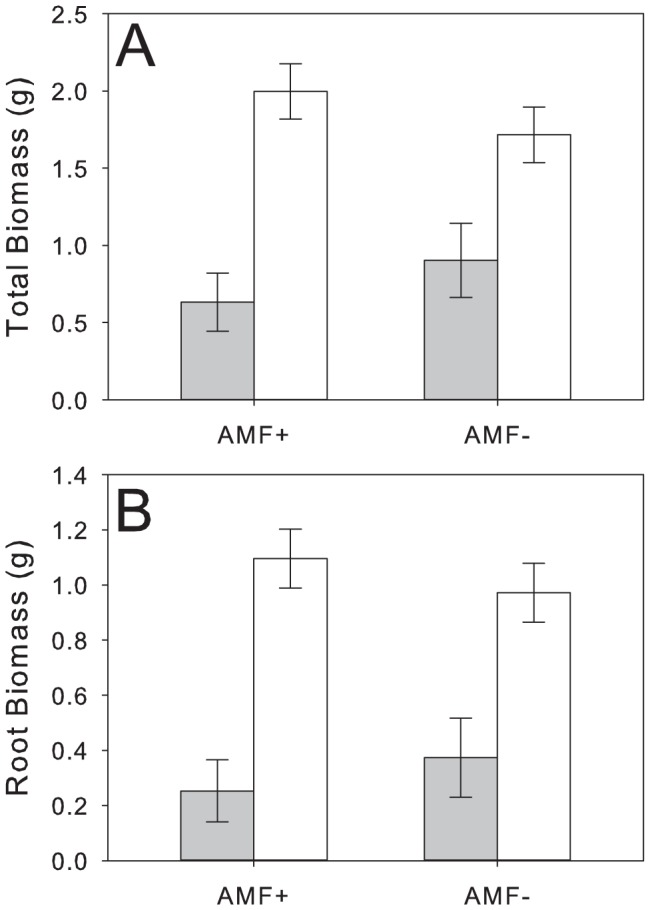
Effects of AM fungal inoculation and vine weevil addition on *P. lanceolata* a) total dry biomass (g) and b) root dry biomass. Grey bars represent the biomass of plants that received 20 vine weevil eggs (thus root herbivory) 3 days after planting, and empty bars represent plants that did not receive vine weevil eggs (and thus experienced no herbivory). AMF+ treatments (along the y-axis) represent plants that were inoculated with an unidentified *Glomus* sp. and *Glomus intraradices* (INVAM #FR121) (sold commercially by Agrauxine, Saint Evarzec, France). Error bars represent ± one standard error. See Table 1 for statistical analysis.

Vine weevil treatment negatively influenced the final total biomass (F_1,30_ = 25.46, *p*<0.0001, Table 1, Fig. 1a) and root biomass (F_1,30_ = 37.63, *p*<0.0001, Table 1, Fig. 1b) of *P. lanceolata*. However, there was no main effect of AM fungi on total plant biomass or root biomass (Table 1).

**Table 1:** ANOVA results for effects of AM fungal inoculation and vine weevil addition on the log-transformed values of *P. lanceolata* biomass.

		Total Biomass			
	*df*	F	*p*	F	*p*
**Block**	1	0.32	0.5761	0.23	0.6333
**AM fungi**	1	0.03	0.8697	0.00	0.9779
**VW**	1	25.46	**<.0001**	37.63	**<.0001**
AM fungi * VW	1	2.06	0.1611	1.48	0.2333
**Error**	30				

ANOVA results for effects of AM fungal inoculation and vine weevil addition on the log-transformed values of *P. lanceolata* total (Fig. 1a) and root (Fig. 1b) biomass. AM fungi refers to the two treatment effect of the presence of AM fungi or sterilized AM fungi within pots, while VW refers to whether 20 Vine Weevil eggs were added to pots three days after planting. Bold values indicate significant effects.

**Figure 2:**
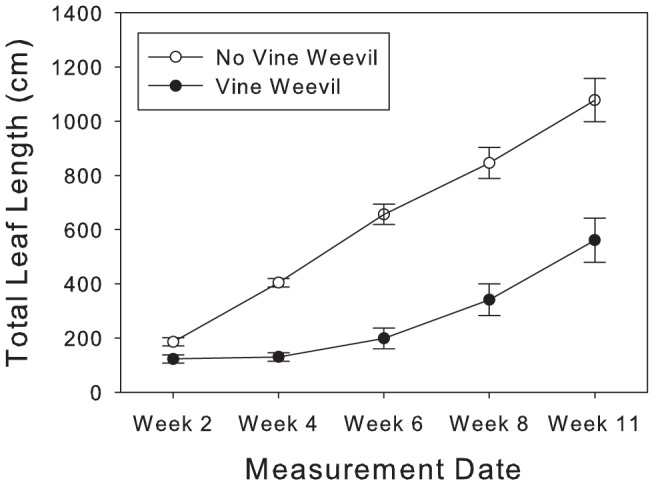
Change in total leaf length (cm) during the experiment. Empty circles represent plants that did not receive vine weevil eggs (and thus never experienced root herbivory) while filled circles represent plants that received 20 vine weevil eggs 3 days after planting resulting in one week of root herbivory ending prior to the Week 2 measurement date. Analyses conducted within this paper show that total leaf length is correlated with total plant size, and thus graphing total leaf length shows the change in plant size in each root herbivory treatment throughout the experiment. Error bars represent ± one standard error.

A significant positive correlation between total plant biomass and total leaf length in plants (r = 0.846, *df* = 35, *p*<0.0001) demonstrated that leaf length was an appropriate proxy for plant biomass, as has been previously demonstrated [32]. The repeated measures ANOVA revealed that *P. lanceolata* growth (as measured using total leaf length) was significantly affected by time (F_4,140_ = 9.32, *p*<0.0001, Figure 2, Table 2), the interaction of time and block (F_4,140_ = 5.31, *p* = 0.0005, Table 2), and the interaction of time and vine weevil (F_4,140_ = 8.97, *p*<0.0001, Table 2), but that the effect of AM fungal inoculation did not vary with time.

**Table 2:** Repeated measures ANOVA on the effect of time and AM fungal and vine weevil treatments upon log of total leaf length of *P. lanceolata*.

	*Df*	F	P
**Time**	4	9.32	**<.0001**
Time*Block	4	5.31	**0.0005**
Time*AM fungi	4	2.15	0.0777
Time*VW	4	8.97	**<.0001**
Time*AM fungi*VW	4	1.83	0.1258
**Error**	140		

Repeated measures ANOVA on the effect of time and AM fungal and vine weevil treatments upon log of total leaf length of *P. lanceolata* (a proxy for total biomass) across five time points spanning the length of the experiment (see Fig. 2). AM fungi refers to the two treatment effect of the presence of AM fungi or sterilized AM fungi within pots, while VW refers to whether 20 Vine Weevil eggs were added to pots three days after planting. Bold numbers indicate significant effects.

### AM fungal colonization

**Figure 3:**
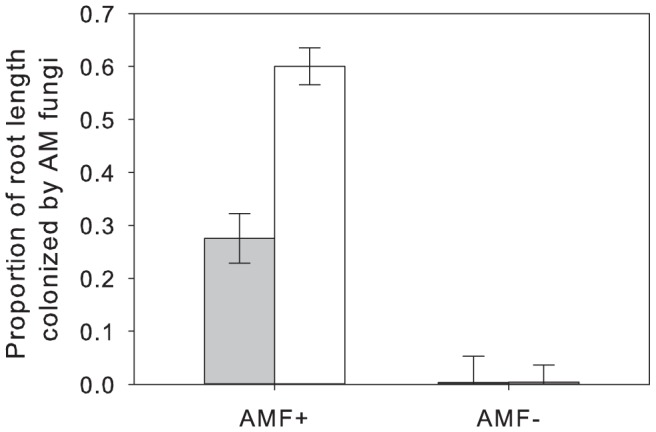
Effects of AM fungal inoculation and vine weevil addition on the proportion of root length colonized by AM fungi AMF+ treatments (along the y-axis) represent plants that were inoculated with an unidentified *Glomus* sp. and *Glomus intraradices* (INVAM #FR121) (sold commercially by Agrauxine, Saint Evarzec, France). Grey bars represent plants which received 20 vine weevil eggs 3 days after planting (and thus herbivory) and empty bars represent plants which never received vine weevil eggs (or experienced no root herbivory). Error bars represent ± one standard error. See Table 3 for statistical analysis.

Sterilization of AM fungal inoculum before addition to pots successfully prevented AM fungal colonization of the roots of *P. lanceolata* in the sterile treatment (F_1,25_ = 325.05, *p*<.0001, Table 3, Fig. 3). Colonization was significantly reduced by vine weevil herbivory (F_1,25_ = 10.08, *p* = 0.004, Table 3, Fig. 3). There was a significant interaction between AM fungal colonization and vine weevil addition (Table 3) as there was no significant difference in colonization in the sterile AM fungi treatment between herbivory treatments (mean = 0 for both treatments), but the vine weevil treatment decreased colonization when both AM fungi and vine weevils were present. No adult vine weevils were recovered from the moats or during the harvest.

**Table 3:** ANCOVA results for the effects of AM fungal inoculation and vine weevil addition on arcsin square root transformed values of the proportion of root length colonized by AM fungi.

	*df*	F	P
**Block**	1	1.54	0.2263
**AM fungi**	1	325.05	**<.0001**
**VW**	1	1.08	**0.0040**
AMF fungi * VW	1	18.43	**0.0002**
**Root Biomass**	1	0.24	0.6297
**Error**	25		

ANCOVA results for the effects of AM fungal inoculation and vine weevil addition on arcsin square root transformed values of the proportion of root length colonized by AM fungi (Fig. 2). AM fungi refers to the two treatment effect of the presence of AM fungi or sterilized AM fungi within pots, while VW refers to whether 20 Vine Weevil eggs were added to pots three days after planting. Root biomass was included as a covariate in order to control for variation in biomass that could influence measures of proportional colonization. Bold numbers indicate significant effects.

### Iridoid glycosides

**Figure 4:**
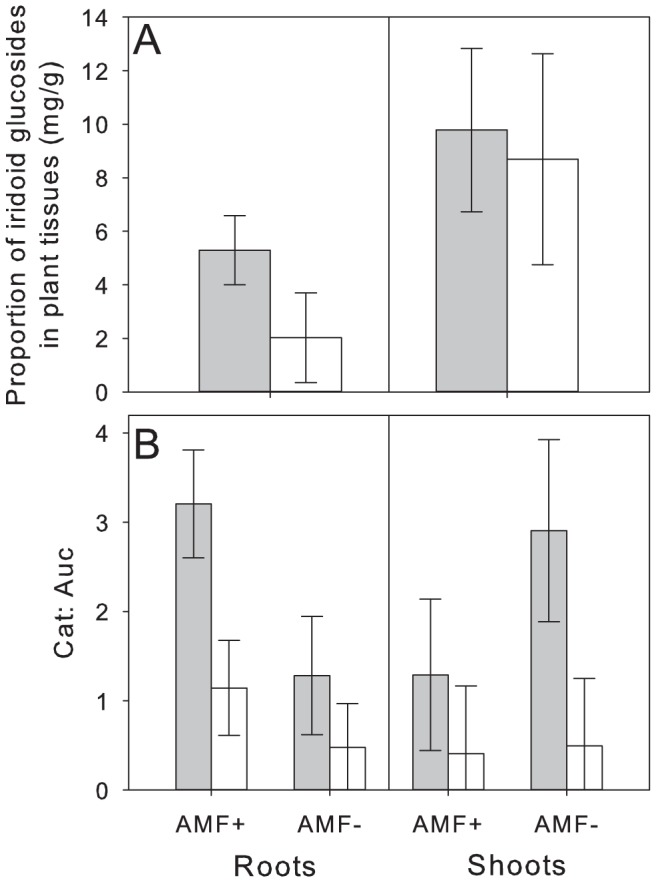
Effects of AM fungal inoculation and vine weevil addition on a) the effect of vine weevils on the concentration of iridoid glycosides (mg/g dry matter) (the primary defensive compounds in *P. lanceolata*) in root and shoot tissues, and b) the interaction between AM fungi and vine weevil presence on the ratio of the proportion of catalpol to the proportion of aucubin in both roots and shoots of *P. lanceolata*. Catalpol and aucubin are the two dominant (and often only) iridoid glucosides present in *P. lanceolata*, and catalpol is derived from aucubin. Catalpol is more toxic to most herbivores than aucubin. Grey bars represent plants which received 20 vine weevil eggs 3 days after planting (and thus root herbivory), while empty bars represent plants that never received vine weevil eggs (nor received root herbivory). The proportions and ratios in the roots of plants are represented on the left of the b graph while proportions and ratios in shoots are represented on the right of the b graph (as denoted by the labels below the b graph). Error bars represent ± one standard error. See Table 4 for statistical analysis.

The presence of vine weevils did not alter the total concentration of iridoid glycosides in *P. lanceolata* root or shoot tissues (Table 4, Fig. 4a). AM fungi did not affect total concentration of iridoid glycosides in either shoots or roots (Table 4). Both the vine weevil treatment (F_1,26_ = 19.90, *p* = 0.0001, Table 4, Fig. 4b), and colonization by AM fungi (F_1,26_ = 5.95, *p* = 0.0219, Table 4, Fig. 4b) increased the ratio of catalpol to aucubin in roots, but these effects were additive, so there was no interaction between the vine weevil and AM fungal treatments. In contrast, the ratio of catalpol to aucubin in shoots only increased in the vine weevil treatment (F_1,29_ = 8.46, *p* = 0.0069, Table 4).

**Table 4:** ANOVA results for effects of AM fungal inoculation and vine weevil addition on the proportion of total iridoid glycosides in root and shoot biomass, and the ratio of catalpol to aucubin in root and shoot biomass.

	Root Total IG			Shoot Total IG			Root Cat∶Auc			Shoot Cat∶Auc		
	df	F	*p*	df	F	*p*	df	F	*p*	df	F	*p*
Block	1	5.06	**0.0328**	1	0.06	0.8063	1	0.05	0.8308	1	0.08	0.7754
AMF	1	0.01	0.9058	1	0.14	0.7094	1	5.95	**0.0219**	1	0.17	0.6832
Vine Weevil	1	2.38	0.1347	1	0.08	0.7759	1	19.90	**0.0001**	1	8.46	**0.0069**
AMF*Vine Weevil	1	0.92	0.3461	1	0.14	0.7063	1	0.85	0.3662	1	0.02	0.8844
**Error**	27			29			26			27		

ANOVA results for effects of AM fungal inoculation and vine weevil addition on the log-transformed values of the proportion of total iridoid glycosides in root biomass, the proportion of total iridoid glycosides in shoot biomass, the ratio of the proportion of catalpol in root biomass to the proportion of aucubin in root biomass (root catalpol∶acubin), and the ratio of the proportion of catalpol in shoot biomass to the proportion of aucubin in shoot biomass (shoot catalpol∶acubin) (see Fig. 3). AM fungi refers to the two treatment effect of the presence of AM fungi or sterilized AM fungi within pots, while VW refers to whether 20 Vine Weevil eggs were added to pots three days after planting. Bold numbers indicate significant effects.

### AM fungal colonization and iridoid glycosides

**Figure 5:**
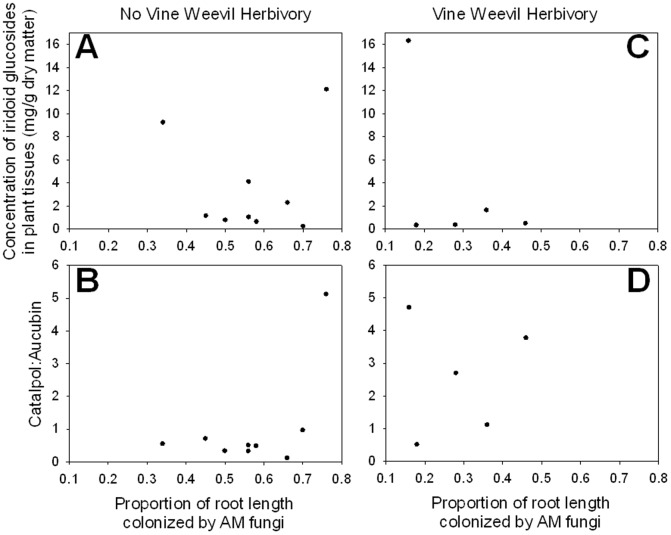
Graphs of the correlations between the proportion of root length colonized by AM fungi and: a) the concentration of iridoid glucosides in plant tissues (mg/g dry matter) in plants that did not receive vine weevil eggs (and thus experienced no vine weevil herbivory). Iridoid glucosides are the primary herbivore defensive compounds in *P. lanceolata*. b) The ratio of catalpol to aucubin in plants that did not receive vine weevil eggs (and thus experienced no vine weevil herbivory). Catalpol and aucubin are the two dominant (and often only) iridoid glucosides present in *P. lanceolata*, and catalpol is derived from aucubin. Catalpol is more toxic to most herbivores than aucubin. c) The concentration of iridoid glucosides in plant tissues (mg/g dry matter) in plants that received 20 vine weevil eggs 3 days after planting (and thus experienced vine weevil herbivory). d) The ratio of catalpol to aucubin in plants that received 20 vine weevil eggs 3 days after planting (and thus experienced vine weevil herbivory). Plants were inoculated with an unidentified *Glomus* sp. and *Glomus intraradices* (INVAM #FR121) (sold commercially by Agrauxine, Saint Evarzec, France).

There was no significant correlation in either the no vine weevil or vine weevil treatment between total iridoid glycoside concentration (r = −0.031, *df* = 9, *p* = 0.9374; r = −0.493, *df* = 5, *p* = 0.3991; Figure 5) and the proportion of root length colonized by AM fungi. There was also no correlation between the ratio of catalpol to aucubin in roots (r = 0.528, *df* = 9, *p* = 0.1442; r = 0.142, *df* = 5, *p* = 0.8193; Figure 5) and the proportion of root length colonized by AM fungi.

## Discussion

As expected, early root herbivory by *O. sulcatus* reduced plant growth. These results are in agreement with multiple studies showing that root herbivory negatively impacts plant growth and fitness [18], although it is notable that even this very brief period of root herbivory early in the plants development continued to have detrimental effects on growth as plants matured. By the end of the experimental period plants may have compensated for root herbivory in above-ground tissues (although we did not measure reproductive output) (Figure 4), however this pattern of compensation did not occur belowground resulting in smaller plants overall.

In contrast to our hypothesis, vine weevil herbivory reduced AM fungal colonization. Although a meta-analysis has shown that shoot herbivory can have a negative influence on AM fungal colonization [48], this is the first study to demonstrate that root herbivory can negatively impact AM fungal colonization. One explanation for the reduction in AM fungal colonization could be that the loss of root tissue led to a loss of root space for AM fungi to colonize. While vine weevil herbivory did reduce root biomass, our analysis of AM fungal colonization controlled for changes in root mass by including root biomass as a covariate, suggesting that the effect of vine weevils on AM fungal colonization extended beyond their effects on colonizable root space. Previous studies have shown that root herbivory has either no effect on AM fungal colonization [13], [14] or increased AM fungal colonization perhaps due to root exudation [15], [16]. Specifically, Currie and colleagues suggested that root exudation lead to an increase in AM fungal available carbon specifically in areas of the root experiencing herbivory [15]. In our study root herbivory was short-lived and therefore the root exudates released by root herbivory may have been limited and therefore did not benefit AM fungi. Other factors that could explain the discrepancy in outcome between this and other studies include differences in host plant species, AM fungal species, root herbivore species used between the various studies, and timing of herbivory during plant development [49]. Currie *et al.* [16] suggested that allowing root herbivory to occur simultaneously with the initiation of AM fungal colonization led to the increase in AM fungal colonization observed in their study. However, given that AM fungi can take up to five weeks to fully colonize roots [50], the application of root herbivores in our study was virtually simultaneous with the introduction of AM fungi. As a result, timing of AM fungal and root herbivore introduction may not explain the effects of root herbivory on AM fungal colonization.

Unlike De Deyn *et al.* [40] we did not find a correlation between the proportion of root length colonized and the concentration of iridoid glycosides in *P. lanceolata* root tissues. The study by De Deyn *et al.* [40] used plants that were the result of several rounds of artificial selection for high and low levels of constitutive iridoids whereas our study used plants collected from a wild population. The lack of a correlation observed in our study may be due to one of two reasons. First, there may exist a weak correlation explaining only a small part of the total variation in AM fungal colonization and iridoid glycoside concentration. As a result, without controlling for genotype within our treatments variation in AM fungal colonization and iridoid glycoside concentrations may have masked this relationship. Second, selection events can select for more than one trait (e.g. [51], [52]), and previous studies of the selected lines examined by De Deyn and colleagues showed that selection on iridoid glycosides also selected for changes in plant biomass and cotyledon size [53]. Thus it is possible that De Deyn and colleagues selected for both changes in iridoid glycoside concentration and root colonization during their selection experiments.

We also hypothesized that vine weevil herbivory would result in increased total concentrations of iridoid glycosides in *P. lanceolota*. However, rather than changes in total iridoid glycoside concentrations, we observed a shift in iridoid glycoside composition in both root and shoot tissues. The only previous study of induction of iridoid glycosides in *P. lanceolata* compared the effects of root and shoot herbivores, and found systemic responses only in plants with constitutively low levels of iridoid glycosides in leaf tissues [42]. Studies in other systems have suggested that root herbivores often induce higher concentrations of secondary compounds in roots (reviewed in [22], [54]). While root herbivory did not cause a systemic increase in the total concentration of iridoid glycosides, it did cause significant differences in the composition of iridoid glycosides in both the roots and shoots. Specifically, vine weevil herbivory increased the ratio of catalpol to aucubin in roots and shoots, suggesting that *P. lanceolata* plants in this study may have deployed a more targeted anti-herbivore response [55]. In particular, increasing concentrations of the more toxic catalpol over a perhaps more costly and/or less effective systemic response (increasing overall concentrations of iridoid glycosides) could reduce future root and shoot herbivory [23], [56]. This response may reflect a selective history of uncorrelated root and shoot herbivory in this population of *P. lanceolata*.

In contrast to the majority of previous studies, root herbivory did not lead to increases in the total concentrations of secondary chemicals in shoot tissues (reviewed in [20], [22], [54]). Aside from differences in secondary chemistry and phylogeny, there is no clear reason why *P. lanceolata* might not systemically increase all secondary compounds in response to root herbivory. This study did not differ greatly in methodology in comparison to previous studies. Root herbivory was brief, however previous studies that showed induced systemic responses followed periods of root herbivory ranging from three days [57] to eight weeks [58]. Our period of herbivory likely falls within this range. Plants in our study were ontogenetically young, only five weeks old at the time of vine weevil addition, however the ontogenetical timing of the application of root herbivores in our study was very similar to the timing of root herbivore addition in the previous study of induction of iridoid glycosides in *P. lanceolata* [42]. As a result, there is no clear explanation for why we did not see a systemic induction of iridoid glycosides concentrations in response to *O. sulcatus* herbivory in *P. lanceolata*.

We also observed shifts in catalpol∶aucubin in the roots of plants hosting AM fungi. AM fungi have been previously shown to contribute to induced relative increases in catalpol in above-ground tissues [23]. Both aucubin and catalpol have been shown to reduce the growth and development of generalist herbivores, but smaller concentrations of catalpol are required to achieve toxic effects ([59] and references therein). In this study we see that AM fungi alter constitutive levels of catalpol in root tissues, but do not influence whether or how iridoid glycoside concentrations are induced by *O. sulcatus* herbivory. This suggests that while there is no interaction between AM fungi and vine weevils on secondary chemical production, AM fungal influenced changes in constitutive levels of catalpol are still likely to negatively impact root herbivores. We would expect that long-term increases in catalpol∶aucubin will select for increased resistance to catalpol or reduced preference for AM fungal colonized plants by *O. sulcatus* larvae.

The combination of the two AM fungal species did not promote host plant growth, but provided other benefits for plant fitness, namely via the promotion of iridoid glycosides in roots following herbivory. This study used two species from one phylogenetic grouping, but there is the possibility that a more diverse AM fungal community or AM fungi from different phylogenetic groups might produce different effects [9].

This is the first study to examine the potential induction of secondary compounds in response to root herbivory and AM fungi in *P. lanceolata*, and showed both shifts in the allocation of plant defense compounds both below- and above-ground in response to root herbivory, and negative effects of root herbivory on AM fungi. Changes to the strength of plant-AM fungal associations and to plant allocation to defence are likely to have far-reaching consequences for soil community diversity and ecosystem function. The picture emerging from this and other studies is of considerable diversity in the nature and strength of plant, AM fungal and herbivore responses to multitrophic interactions. Future research should focus on identifying what circumstances promote predictable ecological responses of plants, AM fungi and herbivores in multitrophic systems.

## Supporting Information

File S1 SAS code for the statistical analyses within the paper.
(DOCX)
