# Supplementary material for: Early Root Herbivory Impairs Arbuscular Mycorrhizal Fungal Colonization and Shifts Defence Allocation in Establishing Plantago lanceolata
Source: PLoS One. 2013 Jun 19;8(6):e66053. doi: 10.1371/journal.pone.0066053 (PMC3686864; doi:10.1371/journal.pone.0066053)
Supplement: File S1 — SAS code for the statistical analyses within the paper. (DOCX) [file pone.0066053.s001.docx]

**Supporting Information**

**SAS Code used to analyze data presented in the manuscript:**

**DATA** vineweevil;

INPUT PlantNumber AMF$ VineWeevil$ Block TotalLeafLength1 TotalLeafLength2 TotalLeafLength3 TotalLeafLength4 TotalLeafLength5 LeafWeight RootWeight TotalBiomass RootAucubin RootCatalpol RootTotalIR ShootAucubin ShootCatalpol ShootTotalIR SectionsScored AMFhyphae Arbuscules Vesicles Spores;

ltotplt=log(**1**+TotalBiomass);

rtsht=LeafWeight/RootWeight;

lrtsht=log(**1**+rtsht);

lRTlIR=log(**1**+RootTotalIR);

lSTlIR=log(**1**+ShootTotalIR);

SCatAuc= ShootCatalpol/ShootAucubin;

lSCatAuc=log(**1**+SCatAuc);

RCatAuc= RootCatalpol/RootAucubin;

lRCatAuc=log(**1**+RCatAuc);

lTlLf1=log(**1**+TotalLeafLength1);

lTlLf2=log(**1**+TotalLeafLength2);

lTlLf3=log(**1**+TotalLeafLength3);

lTlLf4=log(**1**+TotalLeafLength4);

lTlLf5=log(**1**+TotalLeafLength5);

pAMF=AMFhyphae/SectionsScored;

apAMF=arsin(sqrt(pAMF));

**run**;

**proc** **glm** data=vineweevil;

class Soil Block VineW ;

model lRCatAuc lSCatAuc ltotplt lrtsht lRTlIR lSTlIR apAMF =Block Soil VineW Soil*VineW RtWt;

lsmeans Soil VineW Soil*VineW Block /stderr;

means Soil VineW Soil*VineW Block/tukey lines;

**run**;

**proc** **corr** data=vineweevil;

var TlLf5;by VineW;

with Biomass;

**run**;

**proc** **logistic** simple data=vineweevil;

**proc** **glm** data=vineweevil;

class Soil Block VineW;

model lTlLf1 lTlLf2 lTlLf3 lTlLf4 lTlLf5=

Block Soil VineW Soil*VineW /nouni;

repeated time /summary printe;

**run**;
